# Study on Alkali-Activated Prefabricated Building Recycled Concrete Powder for Foamed Lightweight Soils

**DOI:** 10.3390/ma16114167

**Published:** 2023-06-02

**Authors:** Yao Xiao, Zhengguang Wu, Yongfan Gong

**Affiliations:** 1 Suzhou Polytechnic Institute of Agriculture, Suzhou 215008, China; 2 College of Civil Science and Engineering, Yangzhou University, Yangzhou 225127, China

**Keywords:** alkali activated, prefabricated building, foamed lightweight soil, properties, micro-structure

## Abstract

The advantage of a prefabricated building is its ease of construction. Concrete is one of the essential components of prefabricated buildings. A large amount of waste concrete from prefabricated buildings will be produced during the demolition of construction waste. In this paper, foamed lightweight soil is primarily made of concrete waste, a chemical activator, a foaming agent, and a foam stabilizer. The effect of the foam admixture on the wet bulk density, fluidity, dry density, water absorption, and unconfined compressive strength of the material was investigated. Microstructure and composition were measured by SEM and FTIR. The results demonstrated that the wet bulk density is 912.87 kg/m^3^, the fluidity is 174 mm, the water absorption is 23.16%, and the strength is 1.53 MPa, which can meet the requirements of light soil for highway embankment. When the foam content ranges from 55% to 70%, the foam proportion is increased and the material’s wet bulk density is decreased. Excessive foaming also increases the number of open pores, which reduces water absorption. At a higher foam content, there are fewer slurry components and lower strength. This demonstrates that recycled concrete powder did not participate in the reaction while acting as a skeleton in the cementitious material with a micro-aggregate effect. Slag and fly ash reacted with alkali activators and formed C-N-S(A)-H gels to provide strength. The obtained material is a construction material that can be constructed quickly and reduce post-construction settlement.

## 1. Introduction

Prefabricated buildings have the advantages of convenient construction, high construction efficiency, and low costs [1]. It has become a development trend in the construction industry [2,3,4]. For example, the penetration rate of prefabricated buildings has surpassed 70% in Sweden [5,6,7]. Additionally, as concrete is a materials for prefabricated buildings, a large amount of waste concrete building will be produced during the demolition of construction waste. So far, recycled aggregates have been widely used in civil engineering in accordance with some codes for their application of them, such as GB/T25176-2010 “Recycled fine aggregate for concrete and mortar”, GB/T 25177-2010 “Recycled coarse aggregate for concrete and mortar” [8]. However, due to low activity and high porosity, recycled concrete powder has been stockpiled for the long term. As reviewed by some researchers [9,10], recycled concrete powder has been highlighted as a sustainable approach. However, when the dosage is greater than 15%, it has adverse effects on the strength, flowability, and durability of the formed specimen. Therefore, except for a small amount used for low-value products, recycled concrete powder from building waste has not yet been effectively utilized; therefore, it is of great social significance and economic benefit to solve the low activity and realize resource utilization of RCP. Currently, there are recent studies reported on the preparation of alkali-activated recycled concrete powder [11,12,13]. The potential activity of a large amount of SiO_2_ and Al_2_O_3_ in the recycled concrete powder can be improved by alkali activator activation. Hence, as granulated blast furnace slag (GBFS) and fly ash (FA), RCP has been selected by researchers as a raw geopolymer material, which is synthesized by regulating compounds, fineness, and the activation technology of RCP [14,15,16]. The advantages of alkali-activated recycled concrete powder cementitious material include a simple preparation process, no calcination, low energy consumption, low costs, and a large market. It can essentially achieve zero emissions of “three wastes” [17,18,19].

Gong [20] activated recycled concrete powder with NaOH and mixed sodium silicate and discovered that the compressive strength was 18.5 percent higher than that of recycled concrete without an alkali activator. Some researchers [21] found that the activation effect of the NaOH activator is higher than that of the sodium sulfate base activator. Wang [22] revealed that adding an alkali activator promotes the formation of C-S-H and makes the microstructure of recycled micro-powder concrete more compact. The double mixing of the alkali activator and salt activator further promotes the hydration reaction of recycled concrete powder, improves the strength of recycled concrete powder, and produces hydration products; a dense microstructure occurs. Rovnan [23] demonstrated that building waste powder can have pozzolanic properties after being ground to a certain fineness and can react with alkaline components. This property allows it to be used as an active admixture in concrete or as a raw material for the production of geopolymer. Bassani [24] investigated the alkali activation potential of fine particles of recycled construction and demolition waste (CDW) aggregate when mixed with an appropriate alkaline solution. A waste recycled concrete powder is used to produce alternative paving blocks through the alkali activation process.

Foamed lightweight soil is a kind of light material that is prepared by physical method from foaming agent aqueous solution into foam, mixed and stirred with component cementitious materials, water, bubbles, admixtures, additives, etc. in a certain proportion, and hardened by physical and chemical action [25,26,27]. In recent years, due to the advantages of light weight, high strength, good fluidity, convenient construction, and so on, it has been widely used in road engineering, geotechnical engineering, and reinforcement engineering in China [28,29,30]. However, there are several issues with Portland cement as a traditional cementitious in the preparation process of foamed lightweight soil, such as high energy consumption, large pollution, resource waste, high carbon emissions, and so on. Moreover, when cement is used as the raw material of foamed lightweight soil, it is also prone to burst foam and loses its original shape. Therefore, various researchers are focusing on alkali-activated cementitious material as a replacement for cement [31,32,33,34]. Through some research [35,36,37], it was found that the curing system has a significant impact on the properties and microstructure of the recycled concrete powder. In general, an increase in reaction temperature can significantly increase the hydration rate of recycled concrete powder, which has a very beneficial effect on improving the strength of geopolymer.

According to the published literature, alkali-activated recycled concrete powder cementitious materials can achieve similar strength, compatibility, and dry shrinkage properties to cement paste. The production process of alkali-inspired materials consumes less power than cement and is a new type of green material. In this paper, we propose to seek a method for the comprehensive utilization of concrete powder, at the same time, preparing foamed lightweight soil for road engineering. It is suitable for replacement or filling as the main material where conventional materials cannot meet the requirements. It can not only meet the requirements of unit weight but also meet the strength requirements. It can be widely used in fields such as highways, buildings, pipelines, and mine goaf backfilling.

## 2. Materials and Methods

### 2.1. Raw Materials

**Figure 1:**
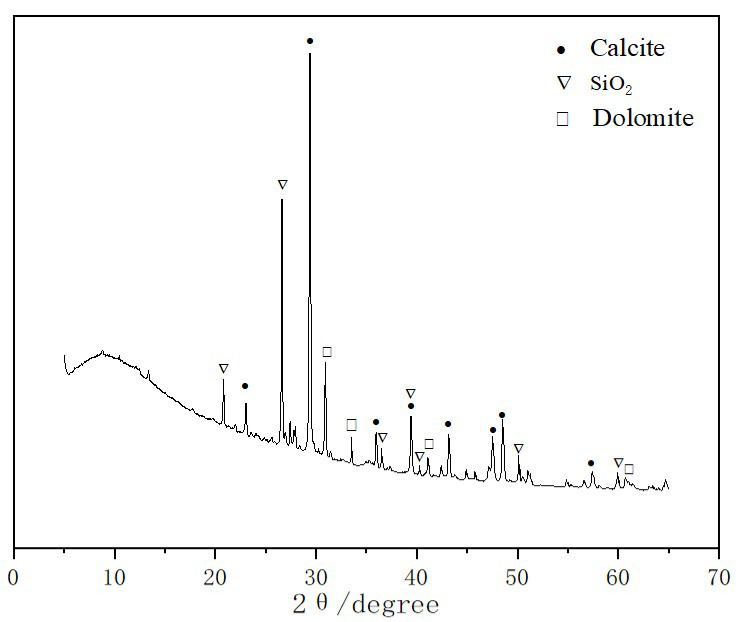
XRD pattern of recycled concrete powder.

**Figure 2:**
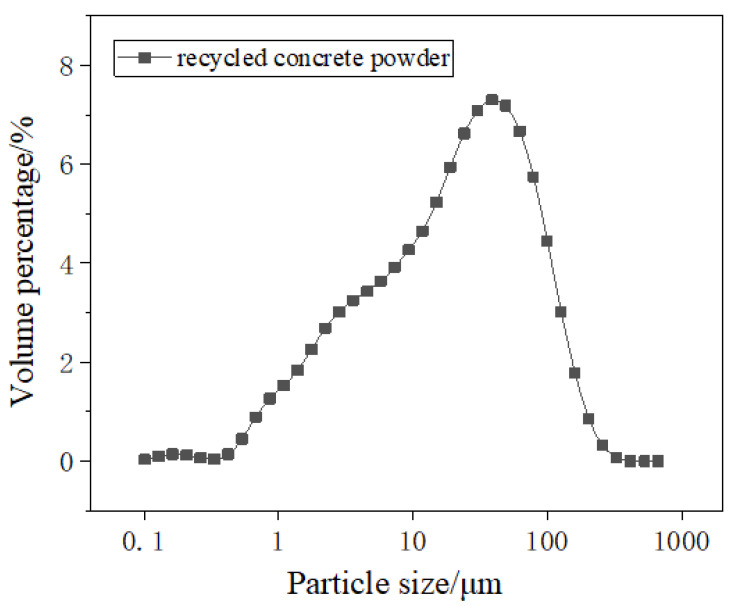
The particle size of recycled concrete powder.

Recycled concrete powder (RCP) is produced from broken waste prefabricated building concrete. It is collected by a dust removal device to manufacture recycled aggregate with a specific surface area of more than 350 m^2^/kg. The slag powder was S95 grade, with an activity index of 96%. The fly ash was of grade II with a low lime content. The chemical compositions of recycled concrete powder, slag, and fly ash are shown in Table 1. As can be seen in Figure 1 and Figure 2, the main components of recycled concrete powder are calcite, silicon dioxide, and dolomite. Among them, calcite is the phase formed after carbonization, while silicon dioxide and dolomite are the phases existing in natural stone and mortar. The medium particle size of the recycled concrete powder is 45 μm.

The alkali activator formulated in this paper is based on sodium hydroxide and industrial water glass as raw materials. The sodium hydroxide was used to reduce the modulus of the industrial water glass solution from 3.3 to 1.4. The dosage of water glass (sodium silicate) was 6%, which was formulated according to the ratio of Na_2_O to cementitious materials. The foaming agent used in this study was sodium dodecyl sulfate (YS-200), with an analytical purity level (AR), and the foam stabilizer is calcium stearate with an analytical purity level (AR).

### 2.2. Design of Mix Proportion

The experiments in this study were guided by the specifications JTG D30-2015 and TJG F1001-2011. It is proposed that the construction wet capacity of foam lightweight soil should be 5.0~11.0 kg/m^3^, and the flow value is 170~190 mm. This experiment began with a single-factor study to investigate the effect of foam admixture on the fluidity, dry density, compressive strength, and water absorption of foamed lightweight soil, to determine the optimal performance of recycled micronized cementitious material foamed lightweight soil. According to the specification, the ratio design of foam lightweight soil can be calculated according to the following steps. Keeping the total mass of the construction wet weight unchanged, which was measured to be 600~1000 kg/m^3^, then gradually adjusting the amount of cementitious material. The components contained 60% recycled concrete powder, 20% slag, and 20% fly ash. Water glass as an activator was measured at 6%. With water to cementitious materials ratio of 0.45 (the total amount of components is 891 kg/m^3^, 794 kg/m^3^, 694 kg/m^3^, 595 kg/m^3^, and 476 kg/m^3^, respectively). After mixing, five mix ratio test groups with a wet density of 600 kg/m^3^–1000 kg/m^3^ were obtained. Calcium stearate is mixed at 2% of the powder mass. The specific mix ratio is shown in Table 2.

### 2.3. Preparation

**Figure 3:**
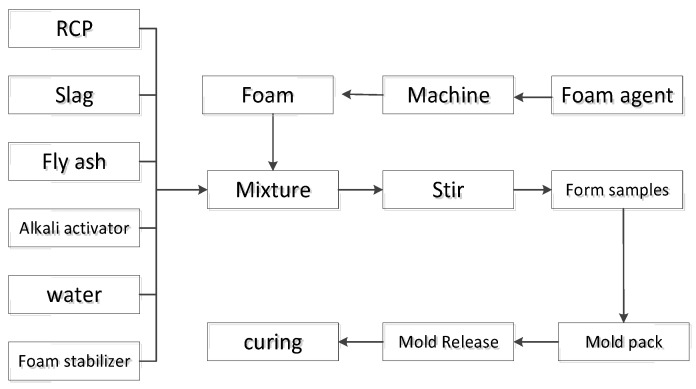
Preparation process of foamed lightweight soil.

First, the foaming agent is mixed with water according to the dilution ratio, and the foaming machine (FP-5) is started to prepare foam. Second, pour the weight of recycled concrete powder, slag powder, fly ash, and calcium stearate into the mixing pot and mix evenly. Third, according to the design requirements of density grade, add corresponding foam, then pour the prepared foam light soil into the corresponding test mold. Lastly, move the entire test mold into a constant temperature cement standard curing box with a relative humidity of >90% and a temperature of 20 °C ± 0.5 °C. Then, remove the formwork 24 h after sample preparation and place it in the curing box again for curing. Upon arrival, take it out for property testing. The Preparation process of foamed lightweight soil has been shown in Figure 3.

### 2.4. Methods

Foamed lightweight soil pastes with varying foam contents were prepared, the w/c was set as 0.45, and a cuboid specimen measuring 40 mm × 40 mm × 160 mm was formed; six samples were tested per group. The wet bulk density weight is the weight per unit volume of freshly mixed foamed lightweight soil. According to the relevant criterion of the specification requirements, its wet bulk density should be less than 1100 kg/m^3^. The fluidity was tested according to GB/T17671-2021, the dry density was tested according to GB/T 5486-2008. Water absorption was tested in accordance with JG/T 266-2011, and unconfined compressive strength in accordance with CECS 249-2008.

The infrared spectrum is primarily used to examine the composition and structure of foamed lightweight soil pastes. Cary 610/670 micro-infrared spectrometer produced by Varian in the United States is used for infrared spectrum testing. Carl Zeiss’ Gemini SEM 300 ZEISS field emission scanning electron microscope system was used.

## 3. Results

### 3.1. Wet Bulk Density

**Figure 4:**
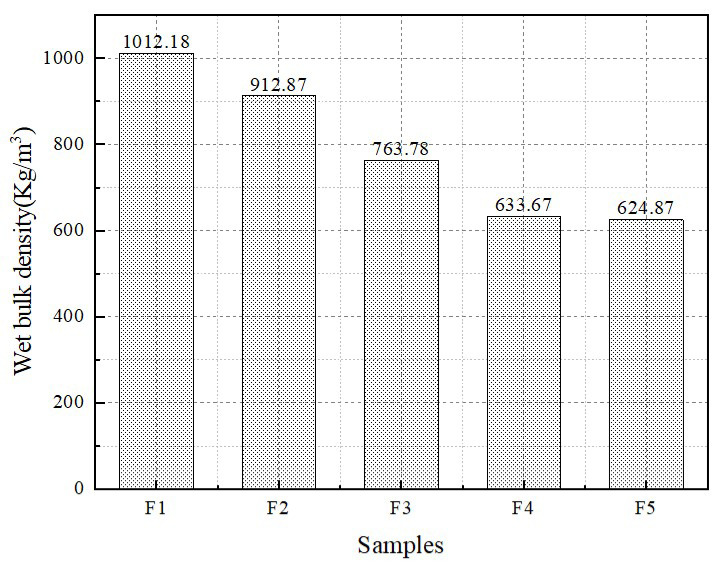
Wet bulk density of foamed lightweight soil.

The wet bulk density of foamed lightweight soil decreases significantly with the increase in foam admixture, as shown in Figure 4. This is due to the use of many bubbles rather than gelling materials, which increases the proportion of generated pores while decreasing the quality of the gelling components. It can be found that the measured wet bulk density is larger compared with the theoretically calculated value. This is because the foam is crushed by the slurry during mixing. The foam soil in a mold was discovered to collapse the phenomenon of F5 to a certain degree, which is due to the addition of excessive foam, resulting in the cementing component being unable to effectively lubricate the foam wall, and the ruptured foam released free water mixed into the slurry, equivalent to improving the water–cement ratio, increasing the wet bulk density of the F5 group.

### 3.2. Fluidity

**Figure 5:**
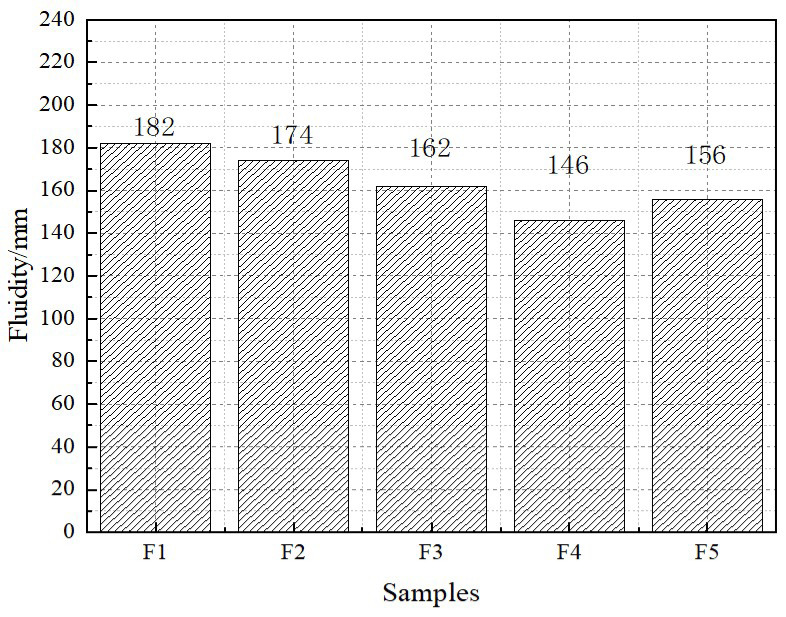
Fluidity of foamed lightweight soil.

Fluidity is an important indicator to measure whether foamed lightweight soil meets the specification requirements. The fluidity should be controlled at 170~190 mm, and the test results are shown in Figure 5. As shown in Figure 5, fluidity decreases significantly with the increase in foam content. This is because the poor fluidity of the foam necessitates slurry wrapping lubrication, and increased production results in insufficient slurry. Calcium stearate has a surface-activating effect and can enhance the liquid wall of the foam. Calcium stearate has hydrophobic properties and can combine with hydroxyl groups to form hydrophobic substances to achieve a bubble retention effect. The overall viscosity of the foam after calcium stearate pacification is large, which reduces the fluidity and requires the cementitious material to play a lubricating role. The alkali-activated slurry has the modifying effect of alkali activation, resulting in an unbalanced charge and a flocculent gel adsorbing a large amount of free water, which will make a low-fluidity slurry.

**Figure 6:**
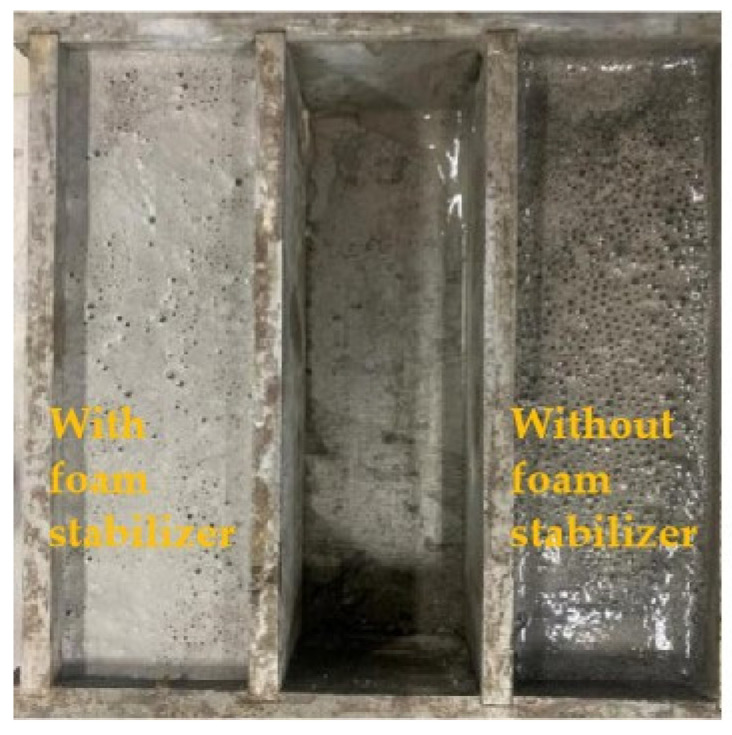
Effect of calcium stearate on bubbles.

Figure 6 shows a freshly mixed recycled powder light soil sample with calcium stearate added to the left and an unadded sample on the right. Many bubbles accumulate on the surface of the foam stabilizer component without the addition of foam stabilizer. This is due to the high flow rate of slurries without foam stabilizers, and the foam is more likely to float to the surface and burst during the stirring process. Despite the addition of calcium stearate to this test sample, excessive foam caused the structural instability of foam in the slurry, the deterioration of bonding performance, and failure to float. In sample F5, many foams burst, the proportion of cementitious materials was increased, and the moisture was released, resulting in a return to fluidity. According to the data, only the F1 and F2 groups meet the criterion requirements.

### 3.3. Dry Density and Water Absorption

**Figure 7:**
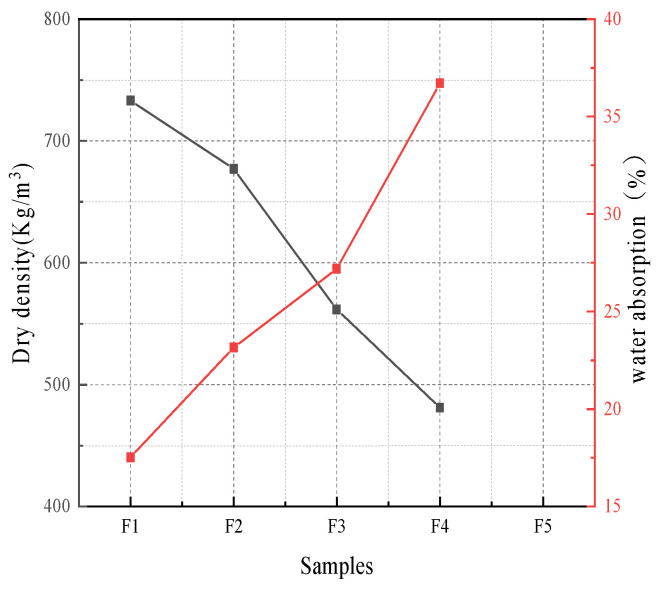
Dry density and water absorption of foamed lightweight soil.

According to the test results shown in Figure 7, the water absorption rate of foamed lightweight soil gradually increases with the increase of the foam content. This is because when the volume content of foam increases, the open pores and connecting pores on its surface also increase, and its ability to absorb water is significantly improved. The increase in water absorption rate gradually accelerates as the foam content increases because when the foam content is too high, the stability of the foam decreases, and small foams easily merge into large foams. Because of the capillary effect, an appropriate pore size is more likely to retain adsorbed water, so the water absorption rate increases linearly when the content is high.

**Figure 8:**
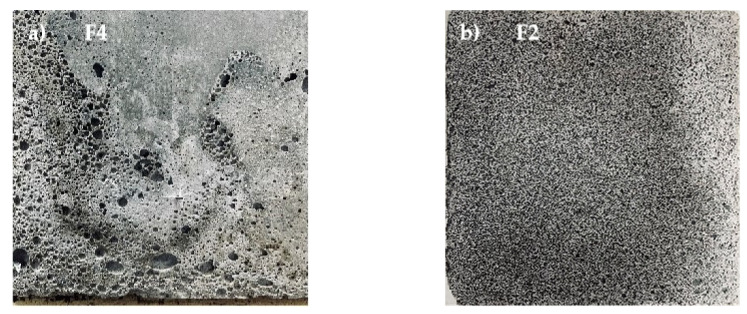
Surface morphology of foamed lightweight soil.

Figure 8 illustrates the surface morphology of samples F4 and F2, respectively. Sample F4 has insufficient slurry, a too-thin foam wall, and a smaller foam burst due to the addition of a large amount of foam. At the same time, some combinations produce foam with larger pore sizes, a more discrete foam distribution, and an uneven structure. The P2 specimen’s slurry and foam are in good condition, and it can be seen that the fine foam is evenly distributed throughout the material, and the sample with this ratio is in better condition. This is because the uniformity of pores improves the molding effect, and better pore structure can improve the strength of the specimen.

### 3.4. Unconfined Compressive Strength

**Figure 9:**
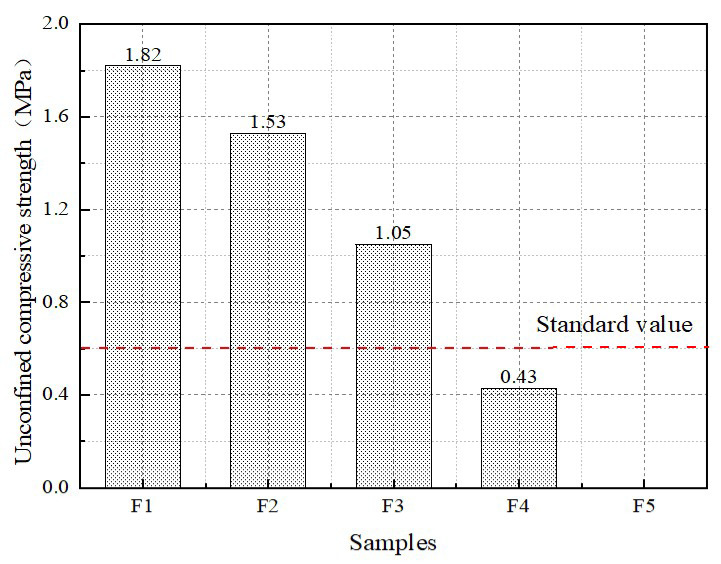
Unconfined compressive strength of foamed lightweight soil.

According to the specification, cubic test blocks with specimen sizes of 100 mm × 100 mm × 100 mm are loaded under unrestricted conditions. As illustrated in Figure 9, the compressive strength gradually decreases with the increase in foam content. F1, F2, F3, and F4 exceed the standard value of 0.6 MPa. This is due to the fact that as the foam content increases, the amount of cementitious material decreases while the porosity increases. This leads to a decrease in the internal load-bearing skeleton of the specimen and a decrease in the strength support part, resulting in a decline in strength. When the foam content is high, the internal foam is unstable and will burst to form large and connected pores, as shown in the image above. Large pores and connected pores cause uneven stress distribution within the material, resulting in weak parts and deteriorating strength. When the foam content is moderate, its distribution is better, and the structure is uniform. Relatively good structural morphology can improve the strength of the material. After testing, all other components can meet the criterion conditions, with the exception of the F5 group, which shows the collapse phenomenon of foamed lightweight soil in the mold.

### 3.5. TG Analysis

**Figure 10:**
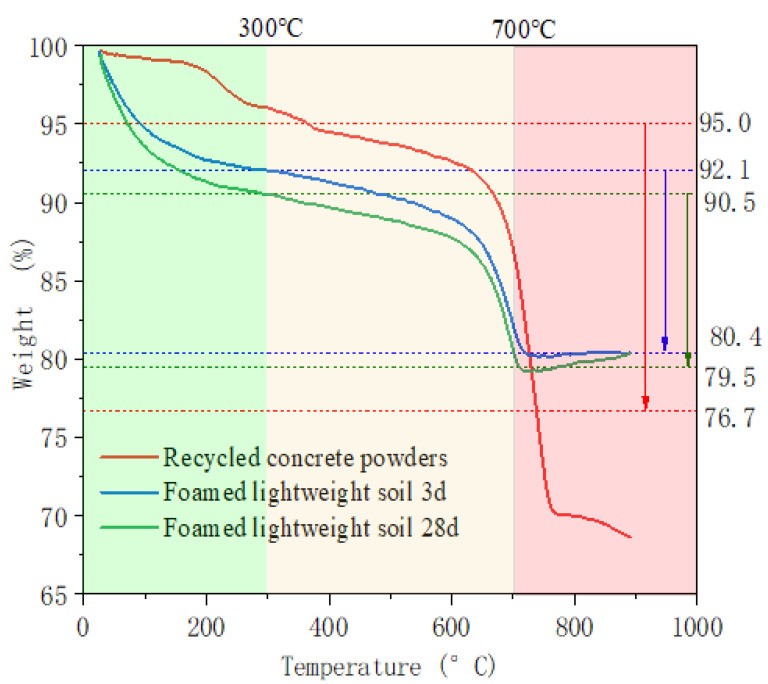
TG analysis of foamed lightweight soil.

According to the TG image of F2 in the Figure 10, at the stage of 20~100 °C, with the increase in age, the weight loss rate at 28 days show a certain increase compared to 3 days. As the time increases, the C-A-S-H gel components increase and the amount of free water adsorbed increases in the foamed lightweight soil. At the stage of 100~300 °C, the weight loss rate is stable and the residual free water gradually decreases. At the stage of 400~500 °C, there was no significant weight loss. However, due to the secondary hydration reaction between Ca(OH)_2_ and the active mixture, residual Ca(OH)_2_ is extremely prone to carbonization into CaCO_3_. Between 600 and 700 °C, the test sample loses a large amount of weight. It is due to the endothermic decomposition of calcite in the sample, which releases CO_2_ and converts to CaO. It can be seen that there is a difference in the decomposition state between the alkali-activated cementitious material and the raw recycled concrete powders. The peak TG value of the cementitious material is around 700 °C, while the peak TG value of the raw recycled concrete powders is around 735 °C. This is becausee the calcite particles formed by carbonization of the cementitious material are very small and can be decomposed at lower temperatures.

### 3.6. Phase Analysis Results of FTIR

**Figure 11:**
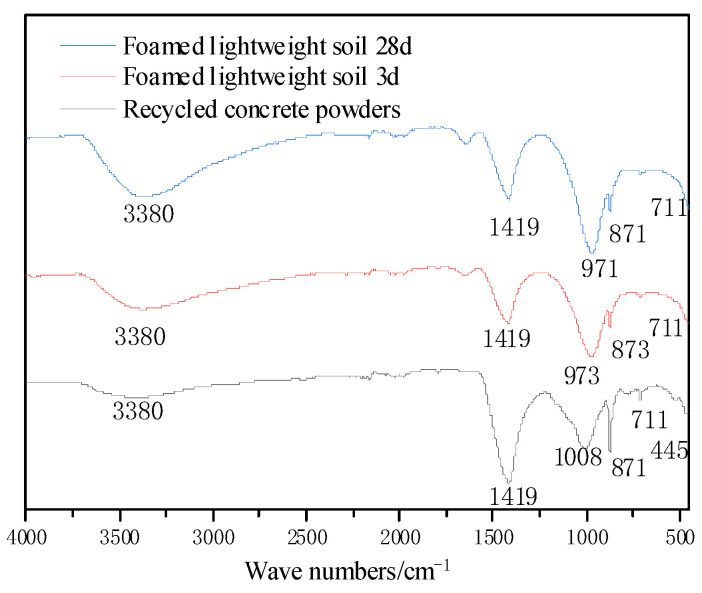
FTIR of foamed lightweight soil.

The FTIR spectra of the foamed lightweight soil of F2 after 3 and 28 days of curing are shown in Figure 11. It is clear that the vibrational absorption peak of the positive silicate Si-O bond at around 445 cm^−1^ smooths out. Around 445 cm^−1^ to 625 cm^−1^ there are the flexural vibration absorption peaks of Si-O-Si or Si-O-Al of chain-shaped and layered silicates. This indicates that under the action of the alkali activator, silicate polymerization occurs, resulting in different degrees of product polymerization. The waveforms of these products overlap on the curve, so the spectrum tends to be flat. The four-coordinate aluminum expansion vibration peak at 873 cm^−1^ weakens with time, and Si-O-R at 979 cm^−1^ weakens and shifts to a lower wave number. Similarly, the carbonate expansion vibration peak at 1419 cm^−1^ weakens and disappears, indicating further depolymerization of these groups with age. However, the foamed lightweight soil of the F2 change trend was similar to that of pure recycled concrete powders. This shows that S-O bonds, Al-O bonds, and C-O bonds are the main products in alkali-activated materials systems. Although C-N-S(A)-H gels covered the original gels, the structure and shape of gels are similar, and the bonding energy becomes stronger.

### 3.7. Microstructure Results of SEM

**Figure 12:**
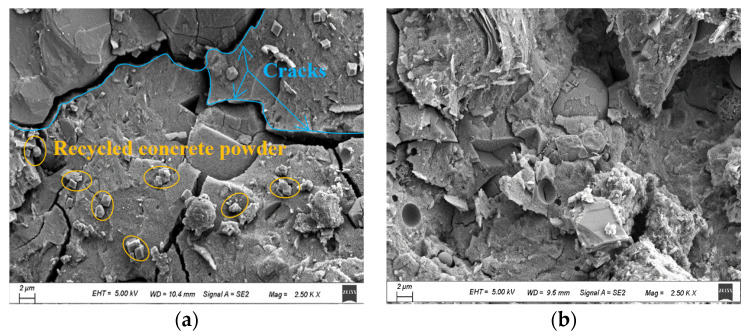
SEM images of foamed lightweight soil: (**a**) 3 d; (**b**) 28 d.

SEM images of the foamed lightweight soil of F2 were observed at different ages. As shown in Figure 12a, the microscopic images of the 3-day-old pastes show more cracks in the surface. This might occur because of the low activity of recycled concrete powders. Simultaneously, the alkali activation reaction of recycled concrete powder, slag, and fly ash cannot be completely reacted in the early stages, which leads to the low strength of foamed lightweight soil. As Figure 12b shows, it is difficult to find wide cracks in the surface of 28-day-old paste. This indicated that in the alkali activating system, the hydration of the foamed lightweight soil can effectively fill gaps between early hydration products. Significantly, the prolongation of time not only improves compactness but also improves the strength of foamed lightweight soil. Therefore, the hydration of alkali-activated foamed lightweight soils is a continuous process and cannot fully react in the early stages.

## 4. Conclusions

The process of foamed lightweight soil preparation using alkali-activated recycled concrete powder has been proposed, and the effect of foam content on the material is revealed in terms of strength, microstructure, and other parameters. Based on our experimental results, the following conclusions and recommendations can be drawn:

1. As alkali-activated raw materials, when the recycled powder, fly ash, and slag occupied 60%, 20%, and 20%, respectively, it can be used to prepare foamed lightweight soil. The wet bulk density is 912.87 kg/m^3^, the fluidity is 174 mm, the water absorption is 23.16%, and the strength is 1.53 MPa, all of which can meet the requirements for light soil for highway embankment.
2. The addition of an appropriate amount of foam stabilizer greatly improves the foam retention of the material. When the foam content ranges from 55% to 70%, it results in an increase in the proportion of foam and a decrease in the material’s wet bulk density. When the foam content is low, the slurry does not wrap the excess foam well, and the material’s fluidity decreases significantly. Too much foam increases the number of open pores and it can easily merge into large foam without stability, which reduces water absorption. At a higher foam content, there are fewer slurry components and lower strength.
3. There are more cracks in the surface of early hydration products, as the reaction progresses, the foamed lightweight soil can effectively fill gaps between early hydration products. However, the hydration of alkali-activated foamed lightweight soils is a continuous process and cannot fully react in the early stages.
4. Compared with the general fill soil or reinforced soil, foamed lightweight soil is convenient for construction without compaction. It is a construction material that can be constructed quickly and reduce post construction settlement, and has higher economic benefits, and is therefore worth spreading and applying.

## Figures and Tables

**Table 1:** Chemical composition of raw materials.

NO.	SiO_2_	CaO	Al_2_O_3_	Fe_2_O_3_	MgO	K_2_O	TiO_2_	Na_2_O	Others
RCP (%)	34.3	42.1	8.2	6.2	4.5	1.2	1.0	1.0	1.5
Slag (%)	30.0	38.1	13.6	0.6	12.5	0.4	0.6	0.3	3.9
Fly ash (%)	61.9	2.4	28.8	2.5	0.8	1.5	1.04	0.3	0.76

**Table 2:** Mix proportions of foamed lightweight soil.

Sample	W/C	Components (kg/m^3^)					$R_{fw}$ (kg/m^3^)
		RCP	Slag	Fly Ash	Water Glass	Foam	
F1	0.45	535	178	178	53.5	1090	1000
F2		476	159	159	47.6	1189	900
F3		416	139	139	41.6	1288	800
F4		357	119	119	35.7	1387	700
F5		278	99	99	29.8	1986	600

## Data Availability

Data are contained within the article.
